# Evidence of potential *Campylobacter jejuni* zooanthroponosis in captive macaque populations

**DOI:** 10.1099/mgen.0.001121

**Published:** 2023-10-25

**Authors:** Xiaoqi Zang, Ben Pascoe, Evangelos Mourkas, Ke Kong, Xinan Jiao, Samuel K. Sheppard, Jinlin Huang

**Affiliations:** ^1^​ Jiangsu Key Laboratory of Zoonosis, Jiangsu Co-Innovation Center for Prevention and Control of Important Animal Infectious Diseases and Zoonoses, Yangzhou University, Yangzhou, PR China; ^2^​ Joint International Research Laboratory of Agriculture and Agri-Product Safety, Ministry of Education of China, Yangzhou University, Yangzhou, PR China; ^3^​ Ineos Oxford Institute for Antimicrobial Research, Department of Biology, University of Oxford, Oxford, UK; ^4^​ Centre for Genomic Pathogen Surveillance, Big Data Institute, University of Oxford, Oxford, UK

**Keywords:** bacterial ecology, *Campylobacter jejuni*, genomics, host adaptation, *Macaca fascicularis*, *Macaca mulatta*

## Abstract

Non-human primates share recent common ancestry with humans and exhibit comparable disease symptoms. Here, we explored the transmission potential of enteric bacterial pathogens in monkeys exhibiting symptoms of recurrent diarrhoea in a biomedical research facility in China. The common zoonotic bacterium *Campylobacter jejuni* was isolated from macaques (*Macaca mulatta* and *Macaca fascicularis*) and compared to isolates from humans and agricultural animals in Asia. Among the monkeys sampled, 5 % (44/973) tested positive for *C. jejuni *, 11 % (5/44) of which displayed diarrhoeal symptoms. Genomic analysis of monkey isolates, and 1254 genomes from various sources in Asia, were used to identify the most likely source of human infection. Monkey and human isolates shared high average nucleotide identity, common MLST clonal complexes and clustered together on a phylogeny. Furthermore, the profiles of putative antimicrobial resistance genes were similar between monkeys and humans. Taken together these findings suggest that housed macaques became infected with *C. jejuni* either directly from humans or via a common contamination source.

## Data Summary

Whole-genome sequencing (WGS) data of *C. jejuni* monkey isolates were submitted to the GenBank database in NCBI (National Center for Biotechnology Information) under the bioproject ID PRJNA1000896 (https://www.ncbi.nlm.nih.gov).

**Impact Statement**

Campylobacteriosis is estimated to account for >160 million annual cases of human gastroenteritis worldwide. Much research is conducted on the animal reservoirs of this zoonosis but humans are seldom considered as an onward infection source for other species (zooanthroponosis). Investigating captive rhesus macaques, which are an important clinical disease model, we sequenced 44 *C *. *jejuni* isolates from symptomatic and asymptomatic monkeys in China. Contrary to other animals that typically harbour host-associated genotypes, macaques were shown to be infected with lineages that are common in human clinical infections in Asia. This is consistent with direct transmission from humans to monkeys or a common infection source. Given the significant genomic similarities between human and non-human primates, this finding has strong implications for understanding how host adaptation and bacterial ecology can influence the spread of important human pathogens.

## Introduction

Agricultural intensification and closer proximity of human and animal populations has increased opportunities for pathogen spill over between hosts [1–4]. This is usually considered in relation to zoonotic transmission from animals to humans, either directly or via contaminated food or the environment [5]. Reverse zoonosis (zooanthroponosis) also occurs, where human pathogens transmit to non-human animals [6, 7]. Both types of host transition present considerable risks but determining the direction (anthropozoonosis vs zooanthroponosis) requires detailed information about the frequency of strains or genotypes in different hosts.

The characterisation of large numbers of isolates from diverse sources and locations, by multilocus sequence typing (MLST) and other methods [7], has revealed considerable genetic differentiation among bacteria from different hosts. Where there is clear association with a particular host or source reservoir it may be possible to identify the origin of human infections, for example, with *Escherichia coli* [8, 9], *Salmonella* [10], *Streptococcus suis* [11], *Staphylococcus* [12] and *Campylobacter* [13, 14]. In *Campylobacter* species and strains vary in the range of hosts from which they have been isolated. For example, some are ecological specialists that are predominantly associated with specific hosts, such as wild bird species, small mammals hosts or reptiles [13, 15–17], while others are ecological generalists that can colonise multiple host species [12, 16]. Where there is frequent host transition, source-associated genetic signatures may be eroded [18], however most extant lineages that infect humans have been linked to agricultural sources [12, 15, 19–23].

*Campylobacter* is among the most common causes of bacterial gastroenteritis and rising antimicrobial resistance has led to it being designated a WHO priority pathogen [24]. Increasing genomic surveillance has been informative for identifying infection sources [25–28] and describing the virulence profiles of isolates. This ranges from strains predominantly linked to asymptomatic infection [26] to those causing multidrug-resistant (MDR) infections [29, 30], and serious post-infection sequelae including Guillain–Barré syndrome (GBS) [31] and irritable bowel disease [32]. Various animal infection models have been used to further understanding of *Campylobacter* virulence, including *Galleria* [33], mouse [30, 34], piglet [35] and monkey systems [36].

The aetiology and symptoms of campylobacteriosis are similar in humans and monkeys. Diarrhoea, abortion and enterocolitis are common in captive rhesus macaques (*Macaca mulatta*) colonies [37–39] and persistent asymptomatic infection can lead to recurrent disease [40]. In this study, we investigate the prevalence of *C. jejuni* infection and asymptomatic carriage in rhesus macaques (*Macaca mulatta*) and cynomolgus macaques (*Macaca fascicularis*) from a primate neurobiology research institute (Suzhou city, China). Using MLST and comparative genomics, the monkey *C. jejuni* population is analysed through comparison with a contextual collection of Asian isolates from human and animal sources. Avergae nucleotide identity (ANI) and antimicrobial resistance (AMR) profiles are analysed to determine the likely origin of *C. jejuni* infections and investigate evidence of a possible host transfer.

## Methods

### Sample collection and monkey housing

Between April 2017 and December 2018, monkeys at the primate neurobiology research institute in Suzhou, China suffered from an outbreak of enterocolitis. This jeopardised animal welfare, disrupted experimental procedures and posed a biosecurity risk. The exact numbers of infected monkeys were not known as the condition was self-limiting in most cases and resolved within 1–2 days. There were too few samples to show significance between the presence of *Campylobacter* being present in diarrhoea samples. A total of 973 faecal samples were collected from rhesus macaques (*Macaca mulatta, n*=231) and cynomolgus macaques (*Macaca fascicularis, n*=742) (Table S1). Monkeys over 1-year-old had been purchased from a laboratory animal company, while younger animals were born and raised in the institute. Monkeys were housed individually or in groups in cages and had no contact with other animal species, except for veterinarians, researchers and breeders. They were fed commercial feed, including apples supplied by a farmers' market in Jiangsu Province, China.

The procedures for collecting faecal samples of experimental monkeys are carried out in accordance with the ethics of the Center of Excellence for Brain Science and Intelligent Technology of the Chinese Academy of Sciences, in the Practice of Assisted Reproduction and Sample Collection in Laboratory Animals of Non-human Primate Research Platform. The faecal sample of keepers and veterinarians who came into contact with the monkeys were collected by the nurse from affiliated hospital of Yangzhou University. The procedures for collecting faecal samples of workers are carried out in accordance with the ethics approval documents from the Medical Ethics Committee of Affiliated Hospital of Yangzhou University. Autoclaved cotton swabs were used to collect the faecal samples, and then the swabs were then placed in Cary-Blair transport medium and transported to the laboratory within 24 h.

### Bacterial culture, DNA extraction and genome sequencing

*C. jejuni* strains were cultured on *Campylobacter* selective agar base plates (modified CCDA, Preston; Oxoid, UK) under microaerophilic conditions (5 % O_2_, 10 % CO_2_ and 85 % N_2_ gas mixture) at 42 ℃ for 48 h, as previously described [41]. These isolates were identified as *C. jejuni* species using polymerase chain reaction (PCR) [41]. Samples positive for *C. jejuni* (*n*=44) were selected for downstream sequencing analysis (Table S1, available in the online version of this article). Genomic DNA was extracted using the TIANamp Bacterial DNA Kit (Tiangen Biotech, Beijing, China), according to manufacturer’s instructions, and quantified on a Nanodrop spectrophotometer prior to normalisation and sequencing. The DNA was fragmented for library preparation and sequenced using an Illumina NovaSeq 6000 (Illumina, USA) at Novogene Institution (Tianjing, China). The resulting reads were assembled into contigs and scaffolds using SOAPdenovo v2.04 (http://soap.genomics.org.cn/Soapdenovo. html). Sequencing statistics for the 44 monkey isolates were analysed by CheckM [42]. Substantially complete genomes are defined between 70–90 %, and the medium contamination is defined between 5–10 % [42]. Genome completeness of 44 monkey isolates ranged from 99.28–99.85 %, with contamination ranged from 0.11 % to ≤3.78 % (Table S2).

### Genome archiving and additional public genomes from Asia

Genomes were assigned STs and clonal complexes using the multilocus sequence typing (MLST) scheme integrated within the pubMLST database (https://pubmlst.org/). Monkey isolates were augmented with 1254 publicly available *C. jejuni* genomes from the pubMLST database collected from across Asia (Table S3). The sequence quality of these genomes were analysed by checkM [42] and pubMLST [43], ensuring all genomes had an assembled length <1.9 Mbp, in <500 contigs, an N95 <900 bp [15], genome completeness ranged from 95–100 %, with contamination estimates from 0.11 % to ≤4.63 % (Table S3). Asian dataset isolates were sampled from various sources; including humans (*n*=838), agricultural animals (chicken, *n*=274; cattle, *n*=24; goat and sheep, *n*=14; duck, *n*=10; goose=15; pig, *n*=1), dog (*n*=3), swan (*n*=6), wide bird (*n*=24), and other sources (*n*=45) (Table S3).

### Core genome characterisation

The average nucleotide identity (ANI) between the isolates from the monkey dataset (Table S4) and the Asia dataset was analysed on a gene-by-gene basis using FastANI v.1.0 [44]. A 95 % ANI threshold was used to determine if genomes belong to the same bacterial species, as previously described [4, 45]. To determine the relatedness of each isolate to one another, a phylogeny was generated using the ParSNP tool (https://github.com/marbl/parsnp) from the Harvest suite [46] based on all single nucleotide polymorphisms (SNPs) identified in each isolate [47]. Phylogenetic trees of monkey isolates only (Fig. S1) and with Asian context isolates were annotated and visualised using Microreact [48].

### Comparison of monkey isolates' pangenome with isolates from other sources

All unique genes present in at least one isolate (the pangenome) were identified by automated annotation using prokka [49] followed by pirate [50]. We defined genes in pirate using a wide range of amino acid percentage sequence identity thresholds for Markov cluster algorithm (MCL) clustering, including 45, 50, 60, 70, 80, 90, 95, 98. Core genes were defined as present in 95 % of the genomes and accessory genes as present in at least one isolate [15].

A pangenome comparison was conducted to compare gene content in isolates from other sources (human, chicken, cattle) in our Asian dataset with our monkey isolates (Table S10). In addition, we compare our monkey-derived isolates with a broader (global) selection of isolates from each source (cattle, chicken and human isolates) (Table S11). A comparable number of isolates (as in Asia dataset; human *n*=838, cattle *n*=24, chicken *n*=274; with an assembled length <1.9 Mbp, assembled in <500 contigs, an N95 <900 bp) were chosen from the pubMLST database (10 times) and compared with our monkey pangenome (pubMLST IDs: Table S12) to identify differences in frequency that monkey accessory genomes are found in isolates from other sources (Table S13–S14).

### Identification of AMR genes and virulence genes

To identify AMR genes in all genomes, a comparison was made with reference nucleotide sequences in the Resfinder Database [51], AMRfinder [52] and CARD (Comprehensive Antibiotic Resistance Database) Database [53]. A positive hit was considered if a gene showed >70 % nucleotide identity with a sequence coverage of at least 70 %. Point mutations associated with antibiotic resistance were identified using PointFinder [54] (Tables S5–S9). Virulence genes were identified by blastn comparison against the Virulence Factor Database (VFDB) [55].

### Antimicrobial susceptibility testing

Phenotypic resistance was assessed using the agar dilution method, as recommended by the Clinical and Laboratory Standards Institute (CLSI) guidelines (VET01-A4, 2013). *C. jejuni* ATCC 33560 was used as the quality control strain. Briefly, colonies were subcultured on *Campylobacter* selective agar base CCDA agar plates for 24 h and then transferred to Mueller–Hinton broth supplemented with 5 % sheep blood (Oxoid, Basingstoke, UK). Known scalar concentrations of antibiotics, including ciprofloxacin (CIP) (0.03–128 µg ml^−1^), erythromycin (ERY) (0.5–256 µg ml^−1^), ampicillin (AMP) (0.5–256 mg ml^−1^), gentamicin (GEN) (0.25–256 µg ml^−1^), chloramphenicol (CHL) (0.25–128 µg ml^−1^), florfenicol (FFC) (0.25–128 µg ml^−1^), clindamycin (CLI) (0.06–128 µg ml^−1^) and tetracycline (TET) (0.25–256 µg ml^−1^), were added to the broth. Strains were classified as resistant (*R*), intermediate (*I*) or susceptible (*S*) according to minimum inhibitory concentration (MIC) breakpoints provided by CLSI.

## Results

### *C. jejuni* can be isolated from asymptomatic and diarrheal monkeys

**Fig. 1.**
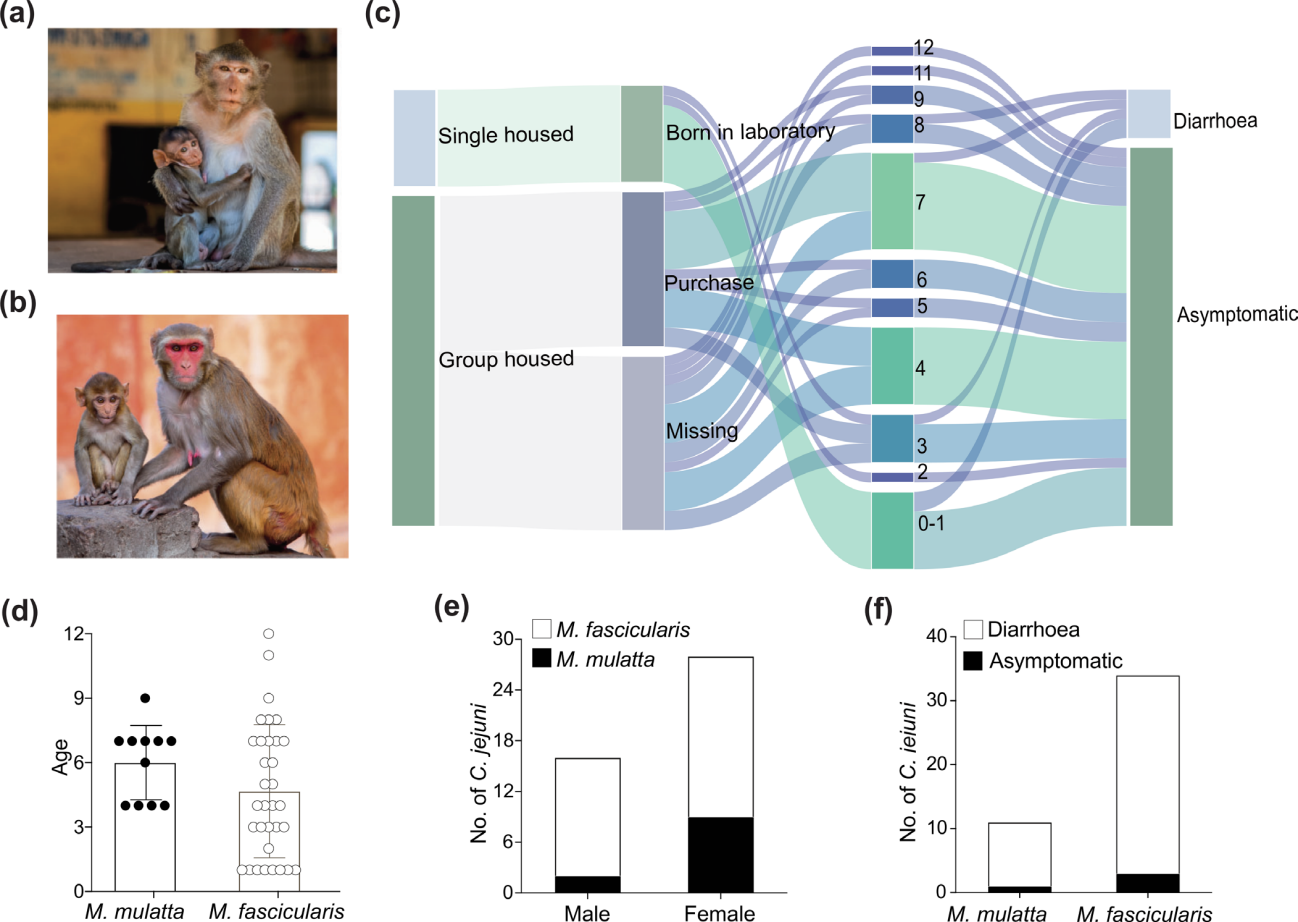
Prevalence of the *C. jejuni* isolates from monkeys. (**a**) *Macaca fascicularis* (cynomolgus monkey). (**b**) *Macaca mulatta* (rhesus monkey). (**c**) Sankey diagram showing the conditions of *C. jejuni *-positive monkeys, including the housing model, monkey birthplace, monkey age, monkey health status, and the number of shared *C. jejuni* clonal complexes. (**d**). Prevalence of *C. jejuni* from the two monkey species. (**e**) Age distribution of *C. jejuni *-positive macaques. (**f**) Sex of *C. jejuni *-positive macaques. (**g**) The physical condition of *C. jejuni *-positive macaques.

Macaques, including *Macaca mulatta* (Rhesus) (Fig. 1a) and *Macaca fascicularis* (cynomolgus) (Fig. 1b), are extensively used as research animal models due to their genetic and physiological similarities to humans [56]. In this study, the aetiology of diarrhoea was investigated in monkeys housed at a primate neurobiology research institute in China. Most of the faecal samples (77.2 %, 751/973) were obtained from group-housed monkeys, while the remaining 22.7 % (222/973) originated from single-housed monkeys (Fig. 1c). In total, 973 faecal samples were collected, comprising 742 samples from *Macaca fascicularis* and 231 samples from *Macaca mulatta*. Among these samples, 4.52 % (44/973) tested positive for *C. jejuni *, with 4.45 % (33/742) and 4.76 % (11/231) positive samples from *Macaca fascicularis* and *Macaca mulatta*, respectively (Fig. 1d, Table S1).

Overall, the most affected age group, among all monkeys, were 4–7-year-old monkeys (23/44, 52.3 %), followed by year 0–3 (14/44, 31.8 %) and year 8–12 (7/44, 15.9 %). The frequency of year 4–7 was significantly higher than year 8–12 (Fisher’s exact test; *P*<0.001). For different monkey species, 7-year-old monkeys (5/11, 45.4 %) were the most affected age group among *C. jejuni* positive *Macaca mulatta*, in contrast 7-year-old monkeys accounted for 15.2 % (5/33) of *C. jejuni* positive *Macaca fascicularis,* but this frequency difference between two monkey species showed no significant (Fisher’s exact test; *P*=0.0228). The most popular affected age group were the 0–1-year-old *Macaca fascicularis* (8/33, 24.2 %) (Fig. 1e).

Female monkeys constituted 63.6 % (28/44) of *C. jejuni *-positive monkeys (Fig. 1f), but this difference was not statistically significant. A total of five isolates (11.3 %, 5/44) were obtained from monkeys with diarrheal symptoms, while the remaining 39 (88.6 %, 44) were obtained from asymptomatic monkeys (Fig. 1g). These diarrheal monkey isolates were obtained from monkeys of various ages, including 0–1 (*n*=2), 3 (*n*=1), 7 (*n*=1) and 8 (*n*=1) years old (Table S1). The two *C. jejuni *-positive monkeys at 0–1 year old were single-housed and born in the laboratory. The remaining three *C. jejuni *-positive monkeys at 3–8 years old were group-housed and purchased from a company. Overall, *C. jejuni* was not detected in faecal samples collected from the breeders, workers in the facility, researchers and veterinary personnel who had daily contact with these monkeys.

### Monkey and human isolates cluster together on a *C. jejuni* phylogeny

**Fig. 2.**
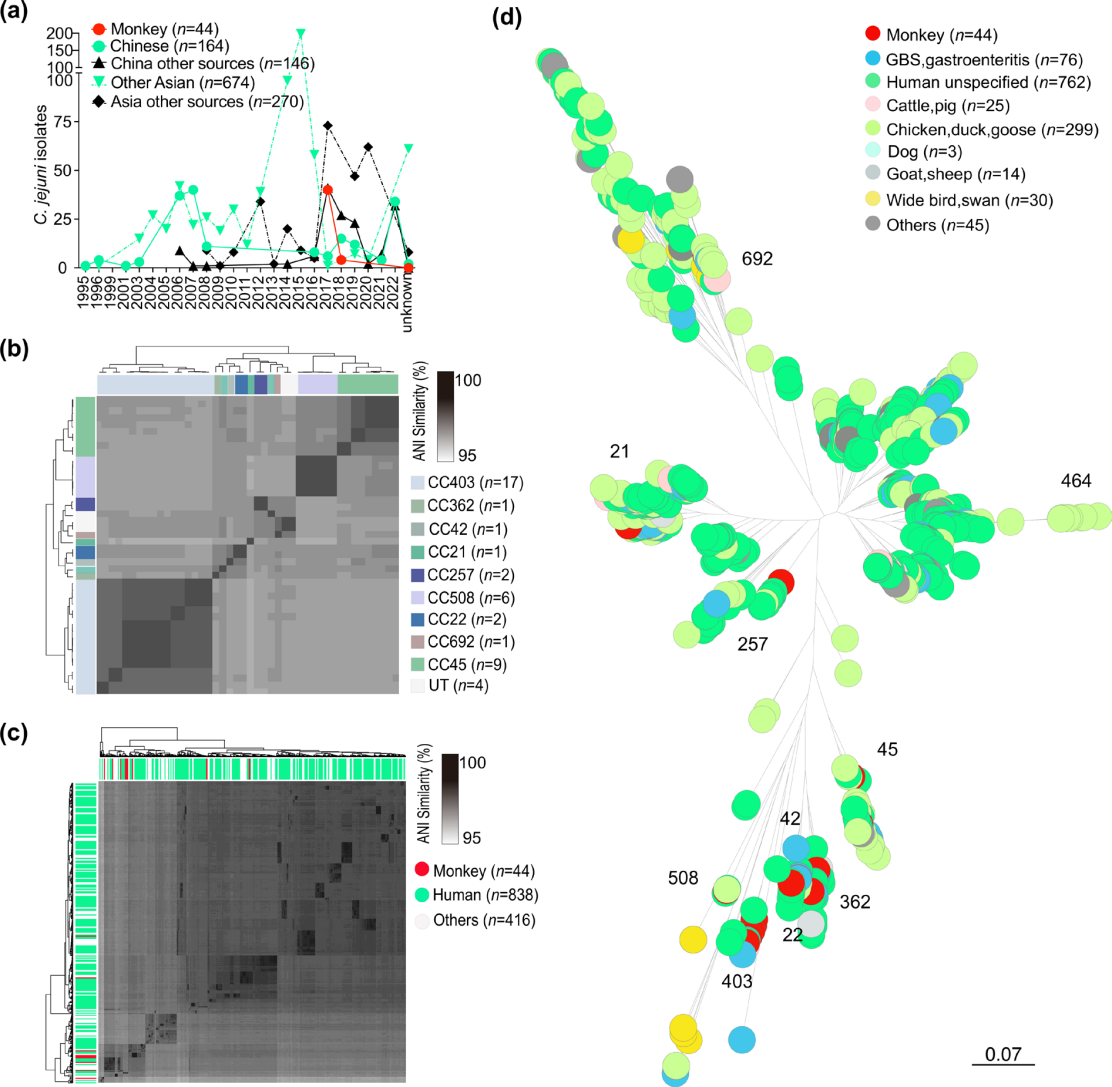
Comparative genomics of isolates from monkeys and human clinical samples in Asia. Comparison of 1298 *C *. *jejuni* isolates indicating (**a**) isolate type and year of isolation. (**b**) Heatmap of pairwise average nucleotide identity (ANI) in the genome of 44 monkey-derived isolates. (**c**) The ANI for each isolate was estimated and averages were compared within and between countries. Isolates from monkeys are marked in red and isolates from humans were marked in green. There is no differentiation between monkey and human isolates based on ANI. (**d**) Phylogenetic tree of 44 *C *. *jejuni* of monkey origin along with 1254 *C *. *jejuni* from multiple sources sampled in Asia. (Monkey isolates visualized separately in Fig. S1.) Visualized on microreact: https://microreact.org/project/n6ZyNeYXjqujfDsyJJ2vpt-fig2-monkey.

To understand the genetic profile of monkey isolates and their relationship with other sources in Asia (Fig. 2a), we generated a core genome similarity matrix (Fig. 2b, c). Among the whole isolates (monkey isolates and Asia dataset), 27.3 % (354/1298) were sampled specifically from China, comprising 44 from macaques, 164 from humans and 146 from other sources (chicken=67, cattle=21, etc.) (Fig. 2a). The remaining 944 isolates were sampled from other Asian countries collected from 1999 to 2020, consisting of 674 from human and 270 from other sources (chicken=207, cattle=3, etc) (Fig. 2a, Table S3). Our dataset represents the known diversity of *C. jejuni* isolates in terms of source and country of origin. Genome-wide ANI values for every possible pair of *C. jejuni* genomes from monkeys ranged from 97.1–99.99 % (Fig. 2b, Table S4), these isolates were clustered by clonal complex.

Monkey isolates did not form a distinct lineage, but clustered together with isolates obtained from humans and other sources (Fig. 2c). This pattern was further supported by the ML phylogenetic tree, which was reconstructed using a gene-by-gene core alignment of genes present in >95 % of all isolates (Fig. 2d). A monkey-only phylogeny was also constructed to ease visualization (Fig. S1). Genome clustered broadly according to ST-clonal complexes, consistent with previous studies (Fig. 2d) [26, 48, 49]. The monkey isolates were assigned to 18 known STs. Among them, ST-403 was the most common ST, representing 15.9 % (7/44) of all isolates (Table S1). A total of 90.9 % (40/44) of monkey isolates belonged to nine known ST-complexes. Two of the remaining four isolates belonged to ST-436 and ST-495, which were not assigned to a clonal complex (CC), while the remaining two isolates were assigned to a new ST-11159 (Table S1). The most prevalent CC was CC403, accounting for 38.6 % (*n*=17) of all isolates (Table S1). This was followed by CC45 (*n*=9), CC508 (*n*=6), CC22 (*n*=2), CC257 (*n*=2), CC21 (*n*=1), CC692 (*n*=1), CC362 (*n*=1) and CC42 (*n*=1) (Fig. 2d, Table S1). The five isolates from diarrhoea monkeys belonged to CC403 (*n*=2), CC508 (*n*=1), CC42 (*n*=1) and CC692 (*n*=1).

### Overlap of lineages between monkeys and human campylobacteriosis cases in Asia

Lineages isolated from monkeys were also found in humans and five other agricultural animal species in Asia. Among the monkey isolates, the most prevalent lineage was the CC403, which has been associated with causing Guillain–Barré syndrome [57]. These isolates clustered with five human isolates from South Asia (India, *n*=1; Bangladesh, *n*=3; Pakistan, *n*=1). We found CC362 and CC22, both associated with GBS [29, 58], were also present in monkey isolates that clustered with human strains from China. CC45 was the second most prevalent clonal complex, isolated from various sources. Monkey isolates from CC45 clustered with human, chicken and goose isolates. Notably, we identified eight CC45 chicken isolates from South Korea in 2017, and two poultry isolates from China in 2017 that also belonged to CC45, indicating the circulation of similar disseminated lineages within East Asia. Another prominent lineage in monkeys CC508 clustered with a chicken isolate from South Korea, one human isolate from India and one human isolate from Israel. These findings indicate that *Campylobacter* genotypes from monkeys are also found in humans and other sources, consistent with recent host transmission.

### *C. jejuni* from monkeys harboured multiple putative AMR genes

Monkey isolates, we screened for the present of AMR genes using three public databases (NCBI AMRfinder Plus, CARD and Resfinder) (Table S5). Most isolates (95.5 %, 42/44) harboured the gene *tet(O*) encoding for resistance against tetracycline, while 90.9 %(40/44) of the isolates harboured the fluoroquinolone resistance single nucleotide polymorphism (SNP), *gyrA* (T86I). Moreover, 43.2 %(19/44) of the isolate genomes harboured the genes *bla*_OXA-193_ (16/44), *bla*_OXA-184_ (2/44) and *bla*_OXA-465_ (1/44), conferring resistance to beta-lactams, and small numbers of isolates harboured aminoglycoside resistance elements (6.8 %, 3/44, *aadE-Cc*). The presence of AMR determinants did not equally contribute to resistance. The *tet(O*) gene was observed in 95.5 %(42/44) of the monkey isolates but only 15 isolates that contained this gene were resistant to tetracycline (Table S5–S6). The concordances of genotypic resistance patterns to phenotypic resistance patterns were also analysed. Six gene patterns from seven isolates (15.9 %) corresponded to six different phenotypic resistance patterns (Table S7). The most common AMR gene pattern was the *tet(O)+gyrA* (T86I) (43.2 %, 19/44) corresponding to 13 phenotypic resistance patterns (Table S6).

### Shared AMR genes between monkey and human isolates from Asia suggests common origin

**Fig. 3.**
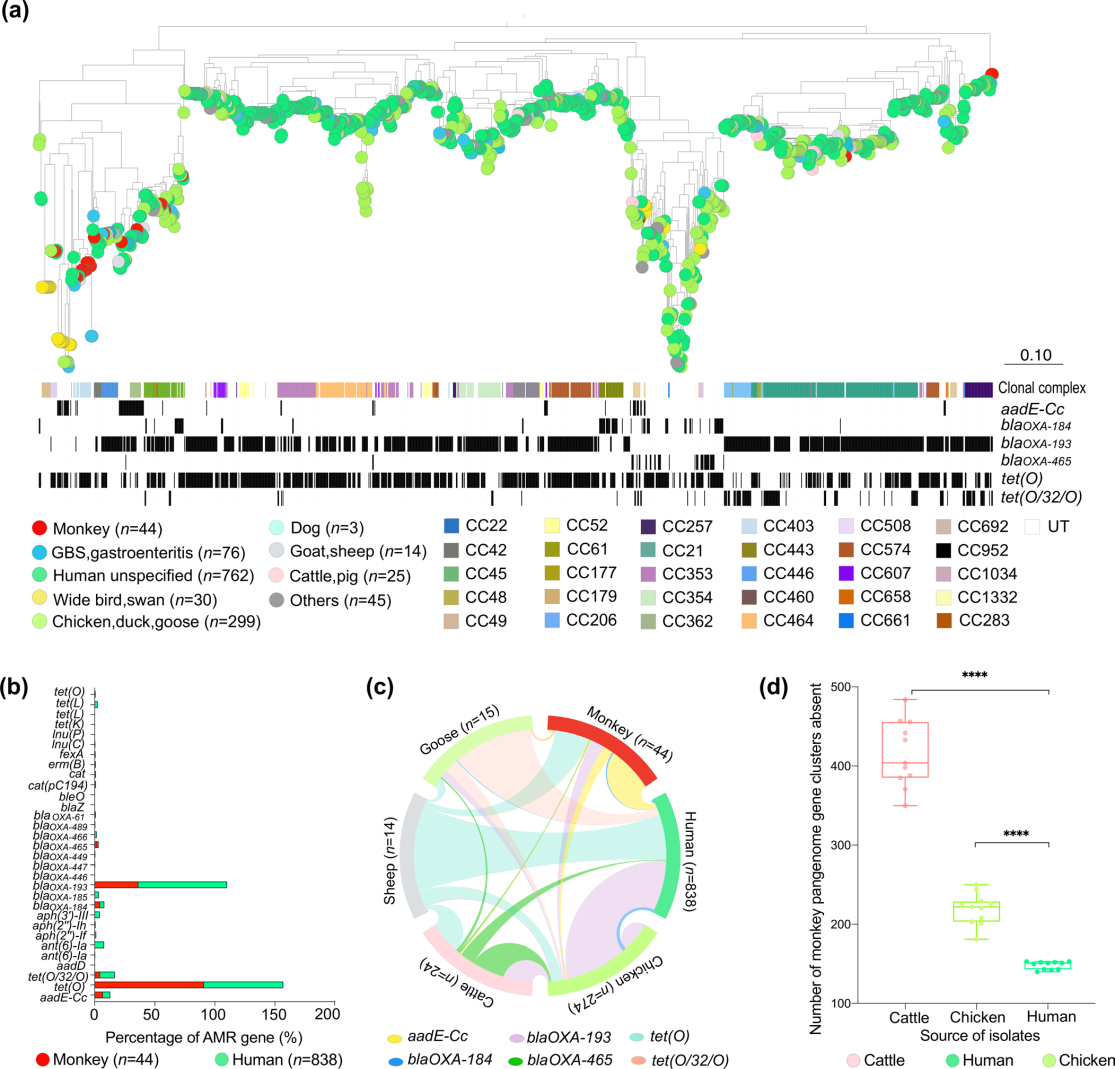
Putative antimicrobial resistance gene and monkey pangenome distribution in *C. jejuni* isolated from human clinical samples in Asia. (**a**) Phylogenetic tree reconstructed using gene-by-gene concatenated alignments of core genes, and the neighbor-joining algorithm for 1298 *C *. *jejuni* isolates indicating the isolate source and clonal complex (CC). https://microreact.org/project/fm8cAUg48ahSV7Dv1NGKnX-fig3monkeyamr. (**b**) The prevalence of AMR genes distributed in the isolates from monkey and humans. (**c**) Distribution of six AMR genes among the *C. jejuni* isolates from various animal sources. (**d**) Comparison of the monkey isolates' pangenome with isolates from other sources (human, chicken, cattle).

The distribution of six antimicrobial resistance genes detected in the genomes of the monkey isolates' dataset was analysed in parallel with human isolates sampled in Asia. Overall, consistency in antimicrobial resistance profiles of *C. jejuni* isolates found in monkeys, humans and other host sources, indicating these monkey isolates could be transmitted between these hosts (Fig. 3a, b, Table S8–S9). Although genomes from monkey isolates harbour less AMR gene variants than human isolates, all the genes detected in the monkey dataset were also present in human isolate genomes. For the 66.7 % (4/6) of the AMR gene detected in monkey isolates, there was no significant difference in the prevalence when compared to those in human isolates (Table S8), these genes included *aadE-Cc*, *tet(O/32/O*), *bla*_OXA-184_ and *bla*_OXA-465_ (Fisher’s exact test; *P*>0.05). The *bla*_OXA-193_ gene was significantly more prevalent in *C. jejuni* from humans (73.6 %, 617/838) than in monkey isolates (36.4 %, 16/44; Fisher’s exact test; *P*<0.001), while the *tet(O*) gene was more prevalent in monkey isolates (90.9 % of monkey isolates, 40/44; 66.7 % of human isolates, 559/838, *P*<0.001).

Monkey isolates also shared AMR genes with the isolates from other agricultural animals (Fig. 3c, Table S9). Similar to human isolates, *bla*_OXA-193_ gene was also statistically significantly prevalent in chicken (55.1 %, 151/274; Fisher’s exact test; *P*<0.05), cattle (70.8 %, 17/24; Fisher’s exact test; *P*<0.05) and sheep isolates (71.4 %, 10/14; Fisher’s exact test; *P*<0.05), compared to isolates from monkeys (36.4 %, 16/44). The *tet(O*) gene was more prevalent in monkey isolates (90.9 %, 40/44) than that in chicken isolates (37.2 %, 102/274; Fisher’s exact test; *P*<0.001) and sheep isolates (64.3 %, 9/14; Fisher’s exact test; *P*<0.05) (Table S9). The similarity in antimicrobial resistance profiles may suggest that strains were transmitted from humans to monkeys.

No significant difference on the frequency of AMR gene pattern was found between monkey isolates and human isolates (Table 1). Nevertheless, for a certain AMR gene pattern, the population structure of monkey isolates can also be found in human isolates (Table 1). Frequency of the AMR gene pattern shared between monkey isolates and human isolates was statistically analysed. Except for *bla*_OXA-193_ *+tet(O*) in human isolates (52.3 %, 406/838; Fisher’s exact test; *P*=0.0078) was significantly more prevalent than that in monkey isolates (27.3 %, 12/44), the frequency of left gene patterns showed no significant difference (Fisher’s exact test; *P*>0.05) between the isolates from two sources (Table 1). Interestingly, the clonal complexes represented in the monkey isolates of a specific AMR gene pattern, were also found in the human isolates of this gene pattern (Table 1). For example, the monkey isolates detected with pattern *bla*_OXA-193_ *+tet(O*) belonging to three known clonal complexes (CC45, CC22, CC21), and these three clonal complexes could also be found in the human isolate population of this AMR gene pattern, suggesting that monkeys are most likely to get contaminated from humans.

**Table 1.** AMR patterns and corresponding clonal complex shared among monkey and human isolates from Asia

Antimicrobial resistance gene patterns	CC	*aadE* *-Cc*	*blaOXA* *-193*	*blaOXA* *-184*	*blaOXA* *-465*	*Tet* *(O)*	*Tet* *(O/32/O)*	Frequency		Frequency_SUM		
								Human	Monkey	Human	Monkey	Fisher_P
*aadE-Cc+bla*_OXA-193_	UT							1.4 % (12/838)	2.3 % (1/44)	2.6 % (22/838)	2.3 % (1/44)	1
	362							1.0 % (8/838)	–			
	353							0.2 % (2/838)	–			
*aadE-Cc +tet(O*)	362							0.1 % (1/838)	2.3 % (1/44)	0.4 % (3/838)	2.3 % (1/44)	0.1854
	UT							0.2 % (2/838)	–			
*aadE-Cc+bla*_OXA-193_+*tet(O*)	UT							0.4 % (3/838)	2.3 % (1/44)	0.5 % (4/838)	2.3 % (1/44)	0.2262
	362							0.1 % (1/838)	–			
*bla*_OXA-184_+*tet(O*)	45							0.2 % (2/838)	4.5 % (2/44)	2.0 % (17/838)	4.5 % (2/44)	0.2440
	443							1.1 % (9/838)	–			
	UT							0.5 % (4/838)	–			
	353							0.2 % (2/838)	–			
*bla*_OXA-193_+*tet(O*)	45							0.6 % (5/838)	15.9 % (7/44)	48.4 % (406/838)	27.3 % (12/44)	0.0078
	22							0.2 % (2/838)	4.5 % (2/44)			
	21							7.5 % (63/838)	2.3 % (1/44)			
	UT							13.0 % (109/838)	4.5 % (2/44)			
	42							0.2 % (2/838)	–			
	48							0.2 % (2/838)	–			
	283							0.1 % (1/838)	–			
	658							0.1 % (1/838)	–			
	952							0.1 % (1/838)	–			
	574							7.2 % (60/838)	–			
	353							5.5 % (46/838)	–			
	464							4.7 % (39/838)	–			
	52							2.4 % (20/838)	–			
	257							1.8 % (15/838)	–			
	354							1.8 % (15/838)	–			
	460							1.1 % (9/838)	–			
	49							0.8 % (7/838)	–			
	607							0.5 % (4/838)	–			
	206							0.6 % (5/838)	–			
*bla*_OXA-193_+*tet(O/32/O*)	257							1.3 % (11/838)	4.5 % (2/44)	6.2 % (52/838)	4.5 % (2/44)	1
	21							2.1 % (18/838)	–			
	206							1.9 % (16/838)	–			
	354							0.4 % (3/838)	–			
	353							0.1 % (1/838)	–			
	49							0.1 % (1/838)	–			
	658							0.1 % (1/838)	–			
	UT							0.1 % (1/838)	–			
*bla*_OXA-465_+*tet(O*)	692							0 % (0/838)	2.3 % (1/44)	0.4 % (3/838)	2.3 % (1/44)	0.1854
	UT							0.2 % (2/838)	–			
	1034							0.1 % (1/838)	–			

### The pangenome of monkey isolates and human isolates are similar

Together, the pangenome of all 44 isolates comprised 2519 gene clusters, with 1420 core genes present in at least 95 % of isolates, representing 56.4 % of the pangenome (Table S10). In total, 94.5 %(2,379/2,519) of the pangenome in monkey isolates were detected in the genomes of Asian isolates, while 94.4–93.9 % of the pangenome could be detected in the randomly selected ten human isolates datasets in pubMLST database (Fig. 3d, Table S11–S13), with the gene coverage over 50 %, gene identity over 50 % (Table S11–S13). The prevalence of monkey-accessory genes in human isolates were significantly lower than those in other sources (*P*<0.0001), the pangenome of monkey isolates and human isolates are similar.

The distribution of virulence genes detected in the genomes of each *C. jejuni* isolate from monkeys and Asia dataset was investigated. The frequency of cytolethal distending toxin (*cdtA*, *cdtB*, *cdtC*) associated genes showed no significant difference in monkey isolates and human isolates (Fisher’s exact test; *P*>0.05). Likewise, putative GBS-associated genes (*wlaN*, *P*=0.0930; *cst-III*, *P*=0.2507), and type IV secretion system protein coding genes (*virB10*, *virB4*, *virB8*, *virB9*, *virD4*; Fisher’s exact test; *P*=0.4022), all showed no significant difference in the frequency in monkey isolates and human isolates (Table S14).

## Discussion

Human activity and globalization of agriculture has increased the transmission of pathogenic bacteria between animal and human species, including non-human primates [59]. Captive macaques are often used in laboratory models for the development of vaccines and therapeutic drugs but are susceptible to idiopathic chronic diarrhoea (ICD). Previous research has indicated that increased transcripts associated with *Campylobacter* are associated with ICD in macaques [60]. However, *Campylobacter* strains have seldom been isolated from non-human primates [61]. In this study we investigated the prevalence and genomic diversity of *Campylobacter* in captive macaques.

*Campylobacter* is a commensal component of the gut microbiota of numerous animal species. Where this is the case, strains isolated in specific hosts typically undergo a period of independent evolution and accumulate host-associated genomic variation. It has previously been shown that signals of host adaptation in the genome of *Campylobacter* transcend those of geographic variation [62, 63], potentially due to dissemination via global food networks. This host segregating genetic variation is the basis for attributing the source of isolates spread from reservoir hosts to humans [24, 64]. Consistent with this, it is possible that captive macaques could harbour host specialist lineages similar to those described for chickens, cattle and wild birds [20, 65–67]. However, several lines of evidence suggest that the strains isolated from macaques in our study represent infection either from humans or from a common human–monkey infection source.

First, the macaque isolates did not form species-specific clusters on a phylogenetic tree. Rather, they clustered with isolates from human infections. Furthermore, comparisons with other isolates collected in Asia (*n*=1,254; between 1995–2022) from more than ten different animal species also showed that isolates from macaques did not cluster by animal species, collection year or country, but by clonal complex. In particular, clonal complexes that are commonly linked to human post-infection sequelae, including CC22, CC362, CC403 [29, 32, 58], that have previously isolated from enteritis patients in Asia [57].

Second, while there was asymptomatic infection in monkeys, there was also symptomatic infection. This variation in disease severity is consistent with human infections [68, 69]. Acute infection in macaques was predominantly caused by CC21 and CC45 [32], two host generalist clonal complexes, previously associated with multiple source reservoirs, including isolates from cattle, chickens and other animals [13, 23, 29]. Nearly a quarter (22.7 %) of the macaque isolates belonged to these two lineages. This is similar to the proportion of these clonal complexes among human infections [69]. This is consistent with a common origin for human and monkey infection strains.

Finally, there was little difference in the prevalence of putative AMR genes detected macaque and human isolates (Table 1). As in published studies [70], the proportion of putative MDR isolates collected from humans in Asia varied but was as high as 90 % in diarrhoeal patients from China (2017–2018). Consistent with this, nearly half (47 %) of the monkey isolates were resistant to three or more antimicrobials. A very high proportion of monkey isolates (95.4 %) were putatively resistant to ciprofloxacin. This is comparable to the levels observed among isolates from human infections in Taiwan (2016–2019; 91.1 %) [71] and Beijing (2017–2018; 94.50 %) [65], and livestock animals in China [30], Europe, and many other countries [72].

Taken together, these findings are consistent with a scenario where macaques became infected with *Campylobacter* either directly from humans or via a common contamination source. Older monkeys that were brought to the facility were screened for some pathogenic species, including *Shigella* spp. and *Salmonella* spp. but not *Campylobacter* spp. Therefore, it is possible that some monkeys were infected on arrival, monkey-to-monkey spread occurred, and symptoms were recorded in some individuals and not others as in human infections [68]. It is also possible that monkeys became infected via food, including local fruits that were sourced from a nearby farm. Finally, the potential role of humans in direct transmission cannot be ruled out as technicians, veterinarians, researchers and breeders were not screened for *Campylobacter *.

The importance zoonotic infections are increasingly acknowledged as a major global threat [73] but zooanthroponoses are less commonly studied. Our study illustrates how enteric bacteria can potentially spread from humans to closely related primates, even in highly controlled research facilities. Whether via direct transmission or through the consumption of contaminated food, this highlights the importance of careful monitoring, and the potential of genomic epidemiology, for animal welfare.

## Supplementary Data

Supplementary material 1

Supplementary material 2

## References

[1] Sheppard SK, Dallas JF, Strachan NJC, MacRae M, McCarthy ND (2009). *Campylobacter* genotyping to determine the source of human infection. Clin Infect Dis.

[2] Sheppard SK, Dallas JF, MacRae M, McCarthy ND, Sproston EL (2009). *Campylobacter* genotypes from food animals, environmental sources and clinical disease in Scotland 2005/6. Int J Food Microbiol.

[3] Alirol E, Getaz L, Stoll B, Chappuis F, Loutan L (2011). Urbanisation and infectious diseases in a globalised world. Lancet Infect Dis.

[4] Mourkas E, Yahara K, Bayliss SC, Calland JK, Johansson H (2022). Host ecology regulates interspecies recombination in bacteria of the genus *Campylobacter*. eLife.

[5] Plowright RK, Parrish CR, McCallum H, Hudson PJ, Ko AI (2017). Pathways to zoonotic spillover. Nat Rev Microbiol.

[6] Lowder BV, Guinane CM, Ben Zakour NL, Weinert LA, Conway-Morris A (2009). Recent human-to-poultry host jump, adaptation, and pandemic spread of *Staphylococcus aureus*. Proc Natl Acad Sci U S A.

[7] Sheppard SK, Guttman DS, Fitzgerald JR (2018). Population genomics of bacterial host adaptation. Nat Rev Genet.

[8] Liu CM, Aziz M, Park DE, Wu Z, Stegger M (2023). Using source-associated mobile genetic elements to identify zoonotic extraintestinal *E. coli* infections. One Health.

[9] Mageiros L, Méric G, Bayliss SC, Pensar J, Pascoe B (2021). Genome evolution and the emergence of pathogenicity in avian *Escherichia coli*. Nat Commun.

[10] Klemm EJ, Gkrania-Klotsas E, Hadfield J, Forbester JL, Harris SR (2016). Emergence of host-adapted *Salmonella* Enteritidis through rapid evolution in an immunocompromised host. Nat Microbiol.

[11] Weinert LA, Chaudhuri RR, Wang J, Peters SE, Corander J (2015). Genomic signatures of human and animal disease in the zoonotic pathogen *Streptococcus suis*. Nat Commun.

[12] Banaszkiewicz S, Calland JK, Mourkas E, Sheppard SK, Pascoe B (2019). Genetic diversity of composite enterotoxigenic *Staphylococcus epidermidis* pathogenicity islands. Genome Biol Evol.

[13] Yahara K, Méric G, Taylor AJ, de Vries SPW, Murray S (2017). Genome-wide association of functional traits linked with *Campylobacter jejuni* survival from farm to fork. Environ Microbiol.

[14] Sheppard SK, Colles FM, McCarthy ND, Strachan NJC, Ogden ID (2011). Niche segregation and genetic structure of *Campylobacter jejuni* populations from wild and agricultural host species. Mol Ecol.

[15] Mourkas E, Taylor AJ, Méric G, Bayliss SC, Pascoe B (2020). Agricultural intensification and the evolution of host specialism in the enteric pathogen *Campylobacter jejuni*. Proc Natl Acad Sci U S A.

[16] Costa D, Iraola G (2019). Pathogenomics of emerging *Campylobacter* species. Clin Microbiol Rev.

[17] Parker CT, Cooper KK, Schiaffino F, Miller WG, Huynh S (2021). Genomic characterization of *Campylobacter jejuni* adapted to the Guinea Pig (Cavia porcellus) host. Front Cell Infect Microbiol.

[18] Dearlove BL, Cody AJ, Pascoe B, Méric G, Wilson DJ (2016). Rapid host switching in generalist *Campylobacter* strains erodes the signal for tracing human infections. ISME J.

[19] Sheppard SK, Cheng L, Méric G, de Haan CPA, Llarena A-K (2014). Cryptic ecology among host generalist *Campylobacter jejuni* in domestic animals. Mol Ecol.

[20] Sheppard SK, Didelot X, Meric G, Torralbo A, Jolley KA (2013). Genome-wide association study identifies vitamin B5 biosynthesis as a host specificity factor in *Campylobacter*. Proc Natl Acad Sci U S A.

[21] Calland JK, Pascoe B, Bayliss SC, Mourkas E, Berthenet E (2021). Quantifying bacterial evolution in the wild: a birthday problem for *Campylobacter* lineages. PLoS Genet.

[22] Jones MA, Marston KL, Woodall CA, Maskell DJ, Linton D (2004). Adaptation of *Campylobacter jejuni* NCTC11168 to high-level colonization of the avian gastrointestinal tract. Infect Immun.

[23] Djeghout B, Bloomfield SJ, Rudder S, Elumogo N, Mather AE (2022). Comparative genomics of *Campylobacter jejuni* from clinical campylobacteriosis stool specimens. Gut Pathog.

[24] Romanescu M, Oprean C, Lombrea A, Badescu B, Teodor A (2023). Current state of knowledge regarding WHO high priority pathogens-resistance mechanisms and proposed solutions through candidates such as essential oils: a systematic review. Int J Mol Sci.

[25] Arning N, Sheppard SK, Bayliss S, Clifton DA, Wilson DJ (2021). Machine learning to predict the source of campylobacteriosis using whole genome data. PLoS Genet.

[26] Mouftah SF, Pascoe B, Calland JK, Mourkas E, Tonkin N (2022). Local accessory gene sharing among Egyptian *Campylobacter* potentially promotes the spread of antimicrobial resistance. Microb Genom.

[27] Berthenet E, Thépault A, Chemaly M, Rivoal K, Ducournau A (2019). Source attribution of *Campylobacter jejuni* shows variable importance of chicken and ruminants reservoirs in non-invasive and invasive French clinical isolates. Sci Rep.

[28] Thépault A, Rose V, Quesne S, Poezevara T, Béven V (2018). Ruminant and chicken: important sources of campylobacteriosis in France despite a variation of source attribution in 2009 and 2015. Sci Rep.

[29] Marotta F, Di Marcantonio L, Janowicz A, Pedonese F, Di Donato G (2020). Genotyping and antibiotic resistance traits in *Campylobacter jejuni* and *coli* from pigs and wild boars in Italy. Front Cell Infect Microbiol.

[30] Zang X, Huang P, Li J, Jiao X, Huang J (2021). Genomic relatedness, antibiotic resistance and virulence traits of *Campylobacter jejuni* HS19 isolates from cattle in China indicate pathogenic potential. Front Microbiol.

[31] Poropatich KO, Walker CLF, Black RE (2010). Quantifying the association between *Campylobacter* infection and Guillain-Barré syndrome: a systematic review. J Health Popul Nutr.

[32] Peters S, Pascoe B, Wu Z, Bayliss SC, Zeng X (2021). *Campylobacter jejuni* genotypes are associated with post-infection irritable bowel syndrome in humans. Commun Biol.

[33] Bojanić K, Acke E, Roe WD, Marshall JC, Cornelius AJ (2020). Comparison of the pathogenic potential of *Campylobacter jejuni*, *C. upsaliensis* and *C. helveticus* and limitations of using larvae of *Galleria mellonella* as an infection model. Pathogens.

[34] Brooks PT, Brakel KA, Bell JA, Bejcek CE, Gilpin T (2017). Transplanted human fecal microbiota enhanced Guillain Barré syndrome autoantibody responses after *Campylobacter jejuni* infection in C57BL/6 mice. Microbiome.

[35] Malik-Kale P, Raphael BH, Parker CT, Joens LA, Klena JD (2007). Characterization of genetically matched isolates of *Campylobacter jejuni* reveals that mutations in genes involved in flagellar biosynthesis alter the organism’s virulence potential. Appl Environ Microbiol.

[36] Hendrickson SM, Thomas A, Prongay K, Haertel AJ, Garzel LM (2022). Reduced infant rhesus macaque growth rates due to environmental enteric dysfunction and association with histopathology in the large intestine. Nat Commun.

[37] Hendrickson SM, Thomas A, Raué H-P, Prongay K, Haertel AJ (2023). *Campylobacter* vaccination reduces diarrheal disease and infant growth stunting among rhesus macaques. Nat Commun.

[38] Sestak K, Merritt CK, Borda J, Saylor E, Schwamberger SR (2003). Infectious agent and immune response characteristics of chronic enterocolitis in captive rhesus macaques. Infect Immun.

[39] Black RE, Levine MM, Clements ML, Hughes TP, Blaser MJ (1988). Experimental *Campylobacter jejuni* infection in humans. J Infect Dis.

[40] Andrade MCR, Gabeira S de O, Abreu-Lopes D, Esteves WTC, Vilardo M de C (2007). Circulation of *Campylobacter* spp. in rhesus monkeys (*Macaca mulatta*) held in captivity: a longitudinal study. Mem Inst Oswaldo Cruz.

[41] Zang X, Tang H, Jiao X, Huang J (2017). Can a visual loop-mediated isothermal amplification assay stand out in different detection methods when monitoring *Campylobacter jejuni* from diverse sources of samples?. Food Control.

[42] Parks DH, Imelfort M, Skennerton CT, Hugenholtz P, Tyson GW (2015). CheckM: assessing the quality of microbial genomes recovered from isolates, single cells, and metagenomes. Genome Res.

[43] Jolley KA, Maiden MCJ (2010). BIGSdb: scalable analysis of bacterial genome variation at the population level. BMC Bioinformatics.

[44] Jain C, Rodriguez-R LM, Phillippy AM, Konstantinidis KT, Aluru S (2018). High throughput ANI analysis of 90K prokaryotic genomes reveals clear species boundaries. Nat Commun.

[45] Goris J, Konstantinidis KT, Klappenbach JA, Coenye T, Vandamme P (2007). DNA-DNA hybridization values and their relationship to whole-genome sequence similarities. Int J Syst Evol Microbiol.

[46] Treangen TJ, Ondov BD, Koren S, Phillippy AM (2014). The harvest suite for rapid core-genome alignment and visualization of thousands of intraspecific microbial genomes. Genome Biol.

[47] Glaize A, Gutierrez-Rodriguez E, Hanning I, Díaz-Sánchez S, Gunter C (2020). Transmission of antimicrobial resistant non-O157 *Escherichia coli* at the interface of animal-fresh produce in sustainable farming environments. Int J Food Microbiol.

[48] Argimón S, Abudahab K, Goater RJE, Fedosejev A, Bhai J (2016). Microreact: visualizing and sharing data for genomic epidemiology and phylogeography. Microb Genom.

[49] Seemann T (2014). Prokka: rapid prokaryotic genome annotation. Bioinformatics.

[50] Bayliss SC, Thorpe HA, Coyle NM, Sheppard SK, Feil EJ (2019). PIRATE: A fast and scalable pangenomics toolbox for clustering diverged orthologues in bacteria. Gigascience.

[51] Zankari E, Hasman H, Cosentino S, Vestergaard M, Rasmussen S (2012). Identification of acquired antimicrobial resistance genes. J Antimicrob Chemother.

[52] Feldgarden M, Brover V, Gonzalez-Escalona N, Frye JG, Haendiges J (2021). AMRFinderPlus and the reference gene catalog facilitate examination of the genomic links among antimicrobial resistance, stress response, and virulence. Sci Rep.

[53] Alcock BP, Raphenya AR, Lau TTY, Tsang KK, Bouchard M (2020). CARD 2020: antibiotic resistome surveillance with the comprehensive antibiotic resistance database. Nucleic Acids Res.

[54] Zankari E, Allesøe R, Joensen KG, Cavaco LM, Lund O (2017). PointFinder: a novel web tool for WGS-based detection of antimicrobial resistance associated with chromosomal point mutations in bacterial pathogens. J Antimicrob Chemother.

[55] Liu B, Zheng D, Jin Q, Chen L, Yang J (2019). VFDB 2019: a comparative pathogenomic platform with an interactive web interface. Nucleic Acids Res.

[56] Vallender EJ, Miller GM (2013). Nonhuman primate models in the genomic era: a paradigm shift. ILAR J.

[57] Islam Z, van Belkum A, Wagenaar JA, Cody AJ, de Boer AG (2009). Comparative genotyping of *Campylobacter jejuni* strains from patients with Guillain-Barré syndrome in Bangladesh. PLoS One.

[58] Nielsen LN, Sheppard SK, McCarthy ND, Maiden MCJ, Ingmer H (2010). MLST clustering of *Campylobacter jejuni* isolates from patients with gastroenteritis, reactive arthritis and Guillain-Barré syndrome. J Appl Microbiol.

[59] Soge OO, No D, Michael KE, Dankoff J, Lane J (2016). Transmission of MDR MRSA between primates, their environment and personnel at a United States primate centre. J Antimicrob Chemother.

[60] Westreich ST, Ardeshir A, Alkan Z, Kable ME, Korf I (2019). Fecal metatranscriptomics of macaques with idiopathic chronic diarrhea reveals altered mucin degradation and fucose utilization. Microbiome.

[61] Ardeshir A, Oslund KL, Ventimiglia F, Yee J, Lerche NW (2013). Idiopathic microscopic colitis of rhesus macaques: quantitative assessment of colonic mucosa: microscopic colitis of rhesus macaques. Anat Rec.

[62] Sheppard SK, Colles F, Richardson J, Cody AJ, Elson R (2010). Host association of *Campylobacter* genotypes transcends geographic variation. Appl Environ Microbiol.

[63] Pascoe B, Méric G, Yahara K, Wimalarathna H, Murray S (2017). Local genes for local bacteria: evidence of allopatry in the genomes of transatlantic *Campylobacter* populations. Mol Ecol.

[64] Thépault A, Méric G, Rivoal K, Pascoe B, Mageiros L (2017). Genome-wide identification of host-segregating epidemiological markers for source attribution in *Campylobacter jejuni*. Appl Environ Microbiol.

[65] Mourkas E, Valdebenito JO, Marsh H, Hitchings MD, Cooper KK (2023). Urbanization spreads antimicrobial resistant enteric pathogens in wild bird microbiomes. bioRxiv.

[66] Colles FM, Ali JS, Sheppard SK, McCarthy ND, Maiden MCJ (2011). *Campylobacter* populations in wild and domesticated Mallard ducks (*Anas platyrhynchos*). Environ Microbiol Rep.

[67] Colles FM, McCarthy ND, Sheppard SK, Layton R, Maiden MCJ (2010). Comparison of *Campylobacter* populations isolated from a free-range broiler flock before and after slaughter. Int J Food Microbiol.

[68] Pascoe B, Schiaffino F, Murray S, Méric G, Bayliss SC (2020). Genomic epidemiology of *Campylobacter jejuni* associated with asymptomatic pediatric infection in the Peruvian Amazon. PLoS Negl Trop Dis.

[69] Nichols GL, Richardson JF, Sheppard SK, Lane C, Sarran C (2012). *Campylobacter* epidemiology: a descriptive study reviewing 1 million cases in England and Wales between 1989 and 2011. BMJ Open.

[70] Zhang P, Zhang X, Liu Y, Jiang J, Shen Z (2020). Multilocus sequence types and antimicrobial resistance of *Campylobacter jejuni* and *C. coli* isolates of human patients From Beijing, China, 2017-2018. Front Microbiol.

[71] Liao Y-S, Chen B-H, Teng R-H, Wang Y-W, Chang J-H (2022). Antimicrobial resistance in *Campylobacter coli* and *Campylobacter jejuni* from human Campylobacteriosis in Taiwan, 2016 to 2019. Antimicrob Agents Chemother.

[72] Sproston EL, Wimalarathna HML, Sheppard SK (2018). Trends in fluoroquinolone resistance in *Campylobacter*. Microb Genom.

[73] Zhang L, Rohr J, Cui R, Xin Y, Han L (2022). Biological invasions facilitate zoonotic disease emergences. Nat Commun.

