# Supplementary figures and images for: Evidence of potential Campylobacter jejuni zooanthroponosis in captive macaque populations
Source: Microb Genom. 2023 Oct 25;9(10):001121. doi: 10.1099/mgen.0.001121 (PMC10634442; doi:10.1099/mgen.0.001121)

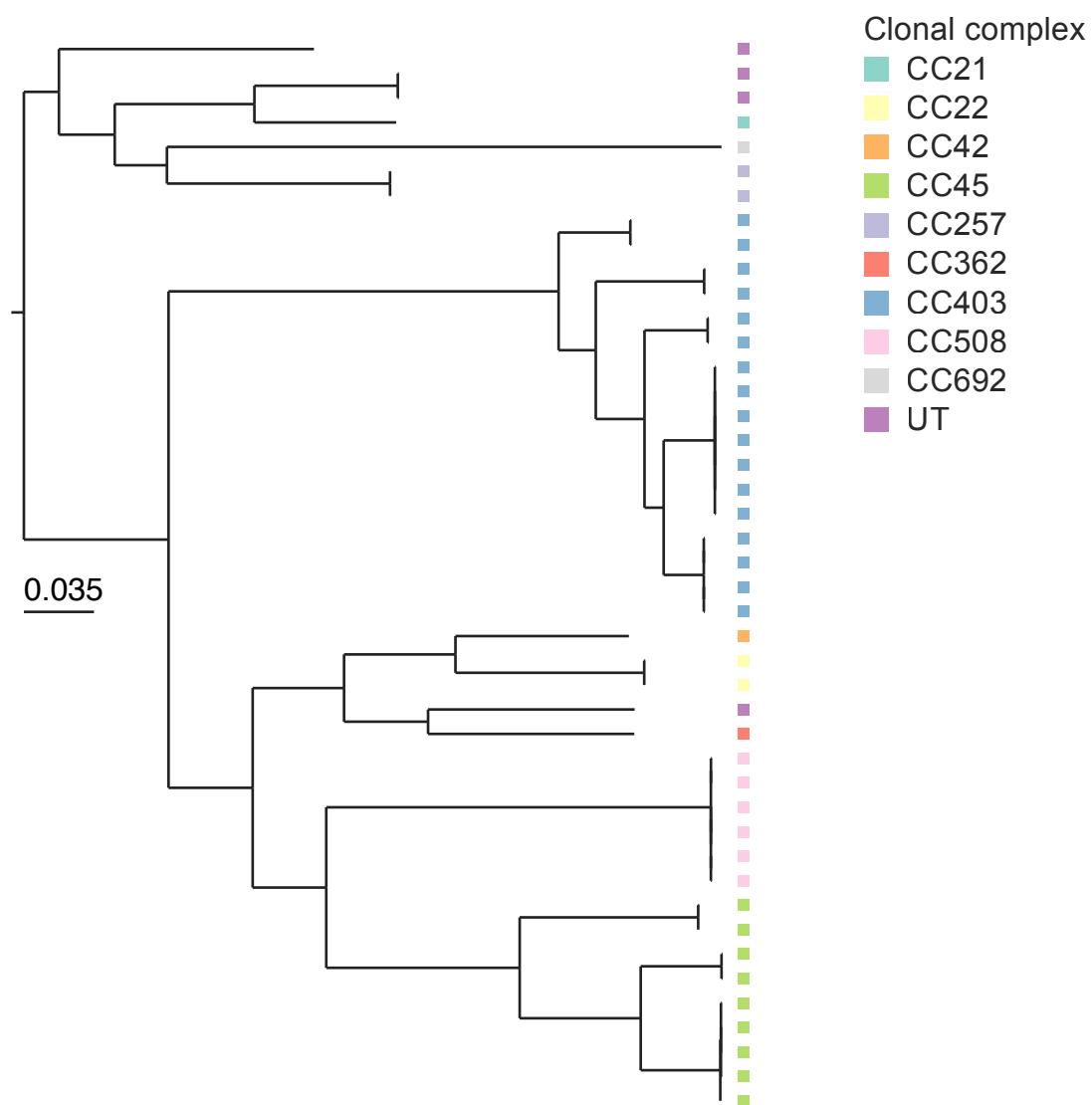

Supplement: Supplementary material 1 [file mgen-9-1121-s001.pdf]
